# Leprosy: clinical and immunopathological characteristics^☆^

**DOI:** 10.1016/j.abd.2021.08.006

**Published:** 2022-04-02

**Authors:** Luis Alberto Ribeiro Froes, Mirian Nacagami Sotto, Maria Angela Bianconcini Trindade

**Affiliations:** a Department of Pathology, Faculty of Medicine, Universidade de São Paulo, São Paulo, SP, Brazil; b Department of Dermatology, Faculty of Medicine, Universidade de São Paulo, São Paulo, SP, Brazil; c LIM56, Hospital das Clínicas, Faculty of Medicine, Universidade de São Paulo, São Paulo, SP, Brazil; d Instituto de Saúde, Secretaria de Estado da Saúde, São Paulo, SP, Brazil

**Keywords:** Immunology, Leprosy, Lymphocyte activation, *Mycobacterium leprae*

## Abstract

Leprosy, a disease caused by *Mycobacterium leprae*, has polymorphic neurocutaneous manifestations strongly correlated with the host immune response. Peripheral neural damage can lead to sensory and motor losses, as well as deformities of the hands and feet. Both innate and acquired immune responses are involved, but the disease has been classically described along a Th1/Th2 spectrum, where the Th1 pole corresponds to the more limited presentations and the Th2 to the multibacillary ones. The aim of this review is to discuss this dichotomy in light of the current knowledge of the cytokines, T helper subpopulations, and regulatory T cells involved in each presentation of leprosy. The text will also address leprosy reactions related to increased inflammatory activity in both limited and multibacillary presentations, leading to exacerbation of chronic signs and symptoms and/or the development of new ones. Despite the efforts of many research groups around the world, there is no standardized serological test/biological marker for diagnosis so far, even in endemic areas, which could contribute to the eradication of leprosy.

## Introduction

Leprosy is a chronic mycobacteriosis with high infectivity and low pathogenicity. It is caused by *Mycobacterium leprae*, whose main host is humans.^1^ Armadillos of the *Dasypus novemcinctus* species can also be naturally infected by *M. leprae*, and there is evidence that armadillos can transmit the disease to humans.^2^ Another bacterium, *Mycobacterium lepromatosis*, discovered in Mexico in 2008, has also been implicated as the etiological agent of leprosy, initially associated with multibacillary forms but later also found in paucibacillary forms.^3^

The availability of free drug treatment for about four decades, a polychemotherapy (PCT) recommended and distributed free of charge by the World Health Organization (WHO), contributed to a change in the natural history of the disease, with a significant increase in cure rates and a substantial decrease in the number of new cases, leading to endemic control in many countries. However, the disease remains an important cause of morbidity in areas with high social vulnerability. In 2019, 202,185 new cases were reported worldwide, of which 27,863 were detected in Brazil.^1^ Despite the polychemotherapy treatment, the disease still has a high disabling potential, particularly when diagnosed at advanced stages, being the main cause of infectious neuropathy in tropical and subtropical countries.

The pathophysiology of leprosy is multifactorial, with genetic, immunological, and environmental aspects determining the individual susceptibility to the bacillus.^1^ Individuals with a weak cell immune response have an intense humoral immune response, with high titers of specific serum antibodies against the bacillus, unable to contain the proliferation of *M. leprae*. These individuals have a high bacillary load, and are called multibacillary. Susceptibility is also influenced by inherited traits, with variable expression of several genes related to the immune response, showing a correlation between the expression of some genes and certain presentations.^4^ Of particular relevance, in lepromatous leprosy and in type 2 leprosy reaction, genes involved in the humoral immune response are expressed at high levels, in particular genes for immunoglobulin receptors or for proteins of the classical complement pathway; in contrast, genes linked to the cell immune response are intensely expressed in the paucibacillary and reverse reaction forms.^5^, ^6^

*M. leprae* genome sequencing from different parts of the world has shown low inter-regional genetic variability, with 99.995% of the genetic material identical between different strains.^7^ This is largely due to the fact that the *M. leprae* genome contains a low percentage of functional genes (less than 50%), with a high percentage of pseudogenes.^8^ However, this finding also suggests that the variability in the clinical presentation of the disease between individuals is mainly due to idiosyncratic factors of the host and not to genetic variations of the microorganism, which has also been demonstrated by studies that correlate some immunogenic variations with certain clinical presentations.^5^

## Immunopathology

The incubation period for leprosy is long, ranging from months to decades, with an estimated average time of 10 years for the lepromatous form and four years for the tuberculoid form. The extremely slow doubling time of the mycobacteria (once every two weeks) makes it more difficult to establish the epidemiological link between a given exposure and disease development. The exact mechanism of leprosy transmission is not known, but the respiratory route is the most widely accepted. There are also other possibilities, such as transmission through insects and inoculation through tattoos, which cannot be completely ruled out.^9^, ^10^, ^11^

It is estimated that over 95% of infected individuals are naturally resistant to *M. leprae*, never developing any signs or symptoms of the disease. Among symptomatic individuals, after the indeterminate phase, the disease manifests itself along a horizontal spectrum proposed by Ridley and Jopling,^12^ based on clinical, bacilloscopic, and histopathological characteristics, containing two poles (tuberculoid and lepromatous) and three intermediate forms (borderline-tuberculoid, borderline-borderline, and borderline-lepromatous). As the clinical presentation progresses from the tuberculoid to the lepromatous pole, a gradual transition occurs from the Th1 to Th2 immune response, according to the model proposed by Modlin.^13^

### The innate response to *M. leprae*

*M. leprae* is a gram-positive, acid-fast bacillus and obligate intracellular parasite, exhibiting marked tropism for phagocytic cells, such as Schwann cells in nerves and macrophages in the skin.^14^ The bacillus is differentiated from other gram-positive and gram-negative bacteria by its lipid-rich cell wall, particularly mycolic acids and phenolic glycolipid-1 (PGL-1). PGL-1 mediates the penetration of the bacillus into macrophages via the C3 fraction of the complement through the CR1, CR3, and CR4 receptors, inducing their phagocytosis. It is a key component for the disease pathogenesis and is involved in the lysosomal escape mechanism,^9^ playing an immunosuppressive role that facilitates the survival of *M. leprae* inside the host cell, the macrophage.

In fact, analyses of cytokines secreted by "naïve" macrophages exposed to PGL-1 and the lipopolysaccharide (LPS) from the outer membrane of gram-negative bacteria show that PGL-1 alone induces a weak production of inflammatory cytokines, such as tumor necrosis factor-alpha (TNF-α), IL-1β and IL-10, while inducing high levels of negative regulatory molecules, such as monocyte chemoattractant protein-1 (MCP-1) and interleukin-1 receptor antagonist (IL-1RA). The microbicidal activity of macrophages involves the production of reactive oxygen and nitrogen species through the NADPH-oxidase complex and nitric oxide, respectively.^10^ A higher expression of induced nitric oxide synthase (iNOS) has been described in tuberculoid skin lesions compared to lepromatous lesions, which can be attributed to the greater Th1-type immune response in paucibacillary leprosy.^10^, ^11^

An important role in the immune response to *M. leprae* has been attributed to toll-like receptors (TLRs) – a type of molecular pattern recognition receptor (PRR) found in monocytes and dendritic cells. TLRs 2 and 4 recognize the leprosy bacillus, leading to the secretion of IL-12 – an interleukin that induces the production of other pro-inflammatory cytokines and the elimination of the bacillus.^12^ A genetic variation, more strongly associated with the lepromatous presentation, was described in the gene encoding TLR2. A functional genetic study has shown that a loss-of-function mutation in this receptor causes less production of IL-12 by macrophages in response to *M. leprae*, suggesting that eventual genetic polymorphisms in this receptor may also affect the natural history of the disease.^15^

The role of the inflammasome in leprosy has also been studied. A component of the innate immune response, the inflammasome is a complex of cytosolic proteins that mediate the inflammatory response through pathogen-associated molecular patterns (PAMPs) and damage-associated molecular patterns (DAMPs), with the latter being responsible for caspase-1 maturation, IL-1β, and IL-18 secretion, in addition to a type of cell death called pyroptosis. A higher expression of inflammasome markers has been reported in lepromatous lesions in comparison to the indeterminate and tuberculoid forms, disclosing the inflammasome inefficiency in controlling *M. leprae* infection.^16^, ^17^

Important in the immune response to *M. leprae*, Schwann cells are able to process and present antigens to CD4 + T cells, triggering an inflammatory process harmful to these cells, which leads to demyelination of the peripheral nerves and neural lesions. *M. leprae* also stimulates the inflammatory response by increasing the Schwann cell sensitivity to the pro-inflammatory cytokine TNF-α, by inducing the transmembrane expression of TNF-α and the TNF-α receptor in these cells.^18^, ^19^

### Acquired response to *M. leprae*

Considering the Th1/Th2 paradigm of leprosy, the cytokine profile found in the skin differs at each pole, with a higher expression of Th1 cytokines (such as IL-7 and IL-15) in people with tuberculoid leprosy and higher expression of Th2 cytokines (such as IL-4, IL-5, IL-10 and transforming growth factor-beta [TGF-β]) in patients with lepromatous leprosy. Peripheral blood analysis of leprosy patients also showed that, after stimulation with recombinant *M. leprae* antigens, there is a predominant induction of Th1 cytokine secretion (IFN-γ, IL-2 and IL-12) in paucibacillary presentations and Th2 cytokines (IL-4, IL-5 and IL-6) in multibacillary presentations.^6^, ^20^

In addition to the classic Th1/Th2 paradigm, studies have shown differences along the spectrum also in relation to Th9, Th17, Th25, and Treg lymphocytes. Th9 lymphocytes form a subpopulation of CD4 + T helper cells, characterized by the induction of the pro-inflammatory interleukins IL-9 and IL-10, potentially amplifying the specific anti-*M. leprae* immune response. In fact, the role of Th9 lymphocytes in leprosy was evaluated in a study that demonstrated higher levels of IL-9 in the tuberculoid pole, explained by the antagonistic function of IL-9 in relation to IL-4 and IL-10, changing the immune response from the Th2 to the Th1 pole.^21^

Similar to Th1 lymphocytes, Th17 lymphocytes produce pro-inflammatory cytokines, most prominently IL-17, which have been associated with the development of reversal reactions in leprosy. IL-17 levels are also elevated in tuberculoid leprosy, where they contribute to the recruitment of inflammatory cells, activation of endothelial cells, and maintenance of the chronic inflammatory process. It is believed that the Th17 response may play a critical role in modulating macrophage activity since IL-17 can induce the production of TNF-α, IL-6, and iNOS, leading to the generation of reactive oxygen species (ROS) that destroy the bacillus.^22^

Th25 lymphocytes are characterized by the expression of IL-4, IL-13, and IL-25 (also called IL-17E), known to amplify the response of Th2 cells and M2 immunomodulatory macrophages. Furthermore, IL-4 and IL-13 are known to inhibit antimicrobial responses and activate B lymphocytes, while IL-25 inhibits cytokine production from Th1 and Th17 responses. The role of Th25 lymphocytes in leprosy has been studied, and it has been observed that their presence occurs at significantly higher concentrations in patients with multibacillary clinical forms, in line with their role in stimulating the Th2 response.^23^

Regulatory T cells (Tregs), phenotypically characterized by the expression of CD4, CD25 (interleukin-2 receptor [IL-2R]), and the transcription factor FOXP3, have also received special attention. They comprise 10% of the CD4 + T cell population in human peripheral blood and are associated with cell response suppression through the production of regulatory/anti-inflammatory cytokines, such as TGF-β and IL-10.^24^ In leprosy, Treg cells are more abundantly found in patients with lepromatous leprosy, thus suggesting that these cells may have a pathogenic role in multibacillary presentations. It has also been demonstrated that Tregs in paucibacillary forms produce more IL-17 (pro-inflammatory cytokine) and less IL-10 (regulatory cytokine), resulting in a microenvironment that favors inflammation and increases tissue damage. Tregs are also known to secrete IL-35, an immunosuppressive cytokine, which inhibits the production of IFN-γ, TNF-α, and IL-2. The increase in IL-35 in leprosy is directly correlated with the patient bacilloscopic index, showing higher levels in the multibacillary forms.^24^

Finally, B cells are also found in the inflammatory response of leprosy lesions, although their role is poorly understood. A study comparing the genetic expression profile in tuberculoid and lepromatous lesions showed that the multibacillary disease was associated with an increase in the plasma cell (CD138+) frequency and an increase in the expression of IL-5 and IgM in the lesions, consistent with the fact that lepromatous leprosy is closer to the Th2 pole.^25^ Contrary to what one might imagine, however, studies have shown a greater presence of CD20+ lymphocytes in paucibacillary forms, a fact attributed to the participation of this cell type in the genesis of the granuloma.^26^

## Immunology of leprosy reactions

Leprosy reactions are episodes of acute hypersensitivity characterized by the worsening of previous lesions or the appearance of new lesions, occurring before, during, or after treatment. More commonly, leprosy reactions occur in multibacillary presentations during the first three months of leprosy specific treatment and currently represent the main disease complication, requiring immediate treatment to prevent neural sequelae. Leprosy reactions can be of two types: reversal reaction (RR) and erythema nodosum leprosum (ENL). RR occurs in approximately one-third of patients with a borderline presentation, whereas ENL occurs in about 50% of patients with lepromatous leprosy and 10% of patients with borderline leprosy, especially in those with borderline lepromatous leprosy.^27^

RR is a type IV hypersensitivity reaction characterized by an exacerbation of the cell immune response against *M. leprae*. If, on the one hand, the reaction leads to the increased elimination of bacilli, on the other hand, it causes an exacerbation of the inflammatory process and the disease symptoms, including neural damage. These lesions are characterized by the presence of a cell infiltrate consisting predominantly of CD4 + T lymphocytes, as well as CD163+ macrophages, with the significant secretion of TNF-α, IFN-γ, IL-2, IL1-beta, IL-6, IL-17, and CXCL-10. The release of TNF-α and IFN-γ, which directly stimulate type Aδ (A Delta) and type C neural fibers, is associated with the pain and swelling that are often seen in RR.^28^

ENL is characterized by a systemic inflammatory process related to extravascular deposition of immune complexes (IC) and neutrophilic exudate. It remains unclear whether IC mediate a type III hypersensitivity reaction or whether their presence is just an epiphenomenon. This reaction occurs in patients with borderline and lepromatous presentations, and it is possible that a large number of antigens and the high production of antibodies, characteristic of Th2 responses, might contribute to the formation of these immune complexes.^29^ ENL is also characterized by the presence of high levels of TNF-α, both in the lesions and in peripheral blood, in addition to IL-2, IL-4, IL-5, IL-6, and IL-10 cytokines, typical of Th2 responses. The successful use of anti-TNF-α therapy with infliximab and etanercept was reported in three patients with ENL, providing additional evidence on the inflammatory role of TNF-α in the process. Similarly, the use of thalidomide in the treatment of erythema nodosum leprosum is based on its capacity to inhibit the production of TNF-α and IL-12 by macrophages.^30^ In addition, thalidomide also affects T lymphocyte function, suppressing its proliferation and stimulating its differentiation into regulatory T lymphocytes (Tregs), with anti-inflammatory action, also inhibiting the nuclear transcription factor Kappa B, involved in the synthesis of several inflammatory cytokines.^31^

## Clinical and histological manifestations

**Figure 1:**
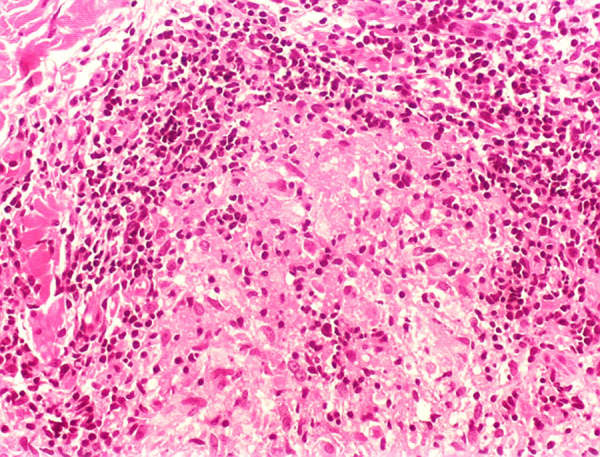
Tuberculoid leprosy. Well-organized epithelioid granuloma, with a dense peripheral halo consisting mainly of lymphocytes. (Hematoxylin & eosin, ×200).

**Figure 2:**
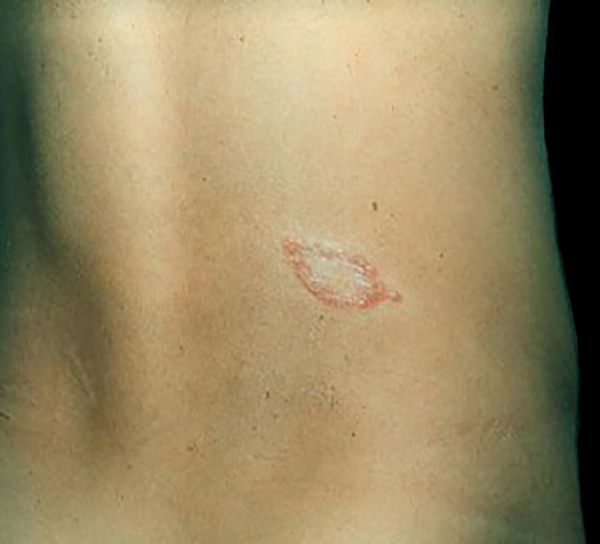
Tuberculoid leprosy. Plaque well demarcated by erythematous papules with a hypochromic center.

The immune response to *M. leprae* determines the histopathological alterations in leprosy, which, in turn, determines the clinical characteristics. Tuberculoid leprosy has well-defined granulomas that reach the epidermis and consist of epithelioid cells, multinucleated giant cells, and macrophages, surrounded by a ring of CD4 + T lymphocytes with the finding of a few or no bacilli (Fig. 1). Clinically, these patients show well-defined erythematous annular plaques (Fig. 2) and loss of sensation, ranging from one to five lesions, and may present with alopecia and/or anhidrosis.^32^, ^33^

**Figure 3:**
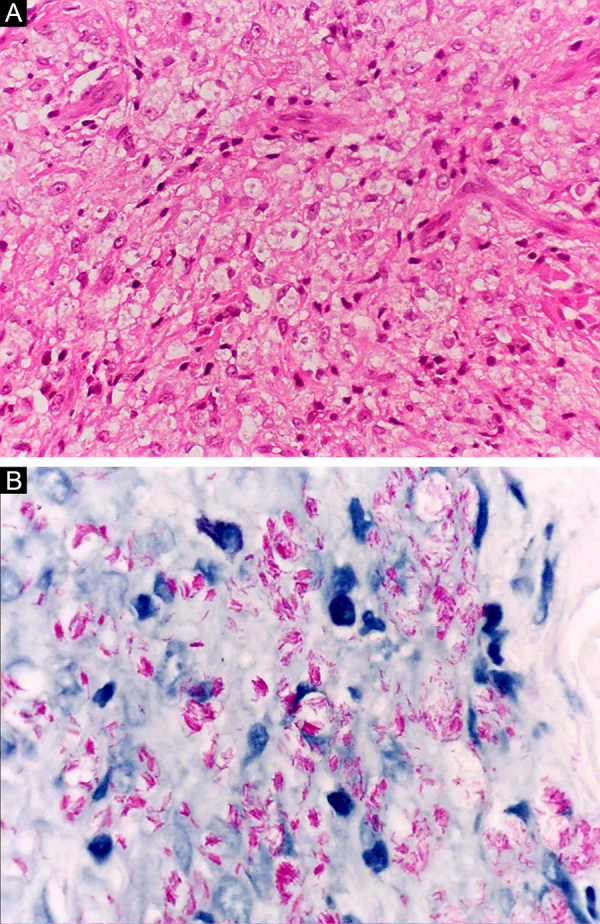
Lepromatous leprosy. (A), Dense dermal cell infiltrate consisting of macrophages with vacuolated cytoplasm (Hematoxylin & eosin, ×400). (B), Numerous isolated acid-fast bacilli forming globi (Faraco, ×1000).

**Figure 4:**
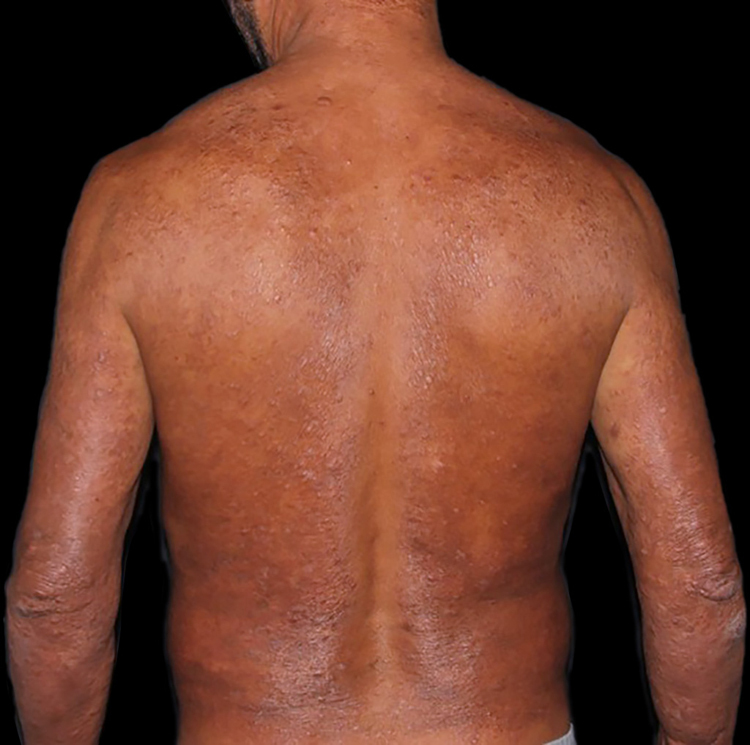
Lepromatous leprosy. Diffuse indurated erythematous-ferruginous plaque, interspersed with macules and papules, sparing the spinal column and axillary regions and predominating on the elbows.

At the other end of the spectrum, individuals with lepromatous leprosy have a large number of lesions, which on histopathological examination reveal vacuolated macrophage granulomas (Virchow cells; Fig. 3a). Macrophage parasitism is abundant, with the formation of globi (Fig. 3b). Clinically, this form is characterized by extensive and multiple bilateral lesions, which may include macules, papules, nodules, and plaques (Fig. 4). In lepromatous lesions, the predilection of *M leprae* for colder regions of the body is clearer, perceived on close examination by the diffuse induration of the face, ears, and/or limbs (elbows and knees). Neural involvement in lepromatous leprosy tends to be slow and multiple, worsening during reactional episodes, while it tends to be unilateral and more acute in tuberculoid leprosy, therefore, progressing earlier to disabilities.^32^, ^33^

**Figure 5:**
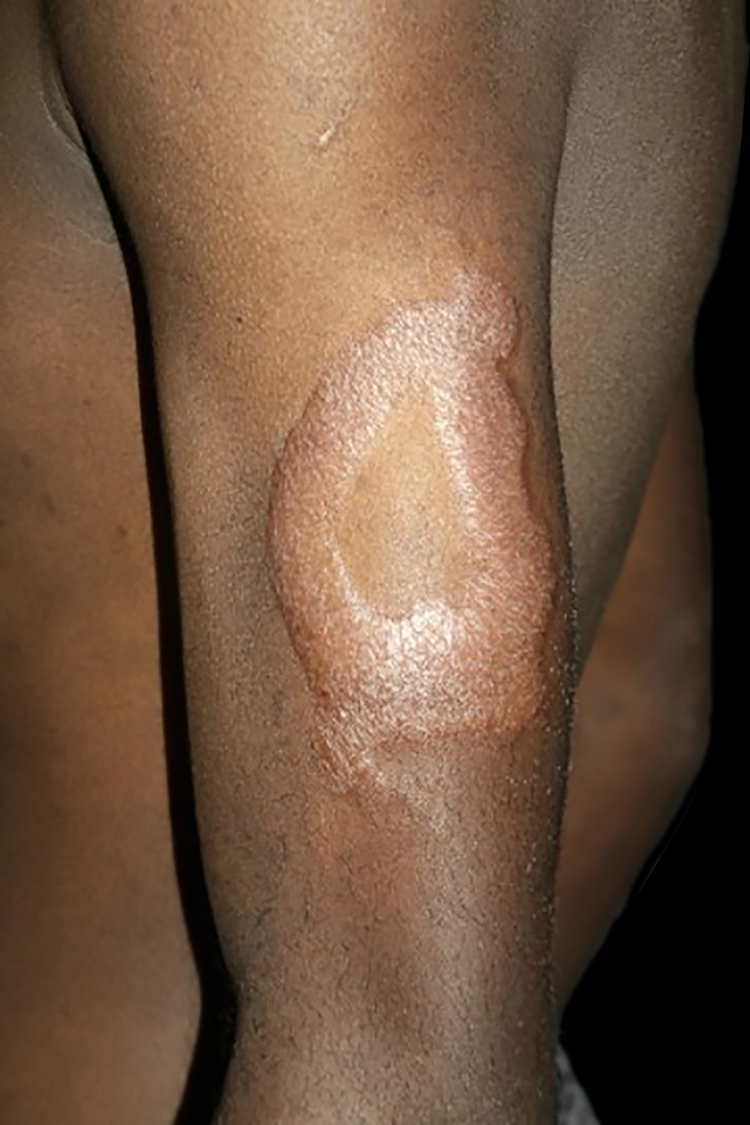
Borderline-tuberculoid leprosy. Indurated-erythematous foveolar plaque, with clear inner and outer edges.

**Figure 6:**
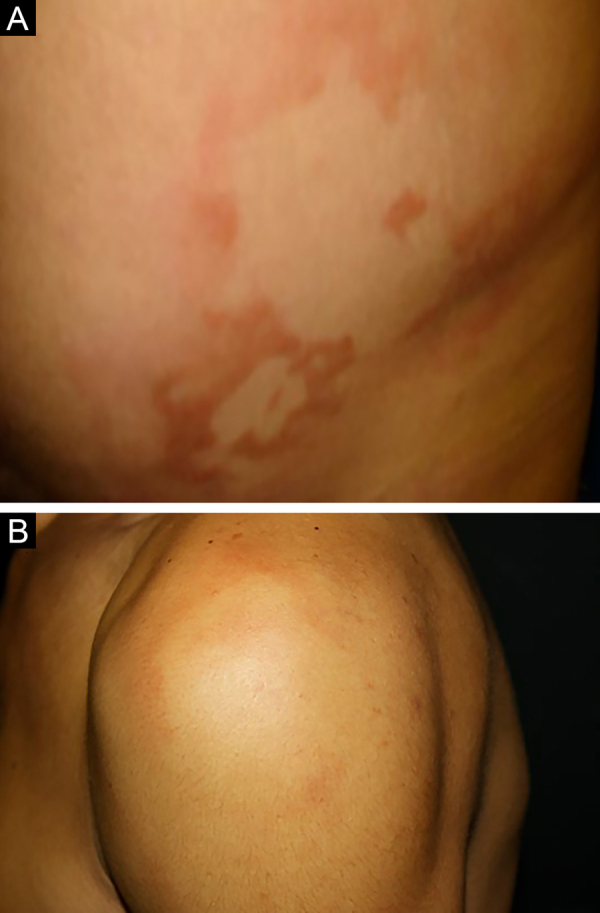
(A), Borderline Leprosy. Well-demarcated erythematous-edematous plaques and papules. (B), Borderline-lepromatous leprosy. Erythematous plaque with a well-defined hypochromic center, and ill-defined outer edges.

Between the tuberculoid (competent cell immune response) and lepromatous (absence of specific cell immune response) poles are the borderline forms. In histopathology, as well as in the clinical presentation, borderline leprosy shows aspects of both tuberculoid and lepromatous leprosy (LL) pole. In individuals with a clinical Borderline-Tuberculoid (BT) presentation, lesions vary in number from five to ten, with larger and less well-defined edges (Fig. 5). Neural trunks are asymmetrically enlarged, causing more severe neuropathy, with neural infiltration on histopathological examination. The inflammatory reaction present in the dermis does not usually reach the epidermis, nor is it as well defined as in the tuberculoid leprosy (TL) form. Borderline-Borderline (BB) individuals have dozens of asymmetrical annular plaques. The classic finding is the borderline lesion, typically annular with poorly delineated outer edges and well-defined inner edges (Fig. 6a). Patients may have peripheral nerve thickening and chronic neuritis. Borderline-Lepromatous (BL) individuals may also exhibit the classic borderline lesions described above, which may be associated with small macules, papules, and nodules of different shapes and sizes (polymorphic; Fig. 6b). There is generalized asymmetric clinical involvement of the peripheral nerves. The tissue inflammatory response in these lesions is similar to that of lepromatous leprosy, but with increased circumscription of the granulomatous macrophage response, a greater number of lymphocytes, and more intense mononuclear infiltrate in nerve filaments and skin appendages (hair follicles, sweat glands).^32^, ^33^

**Figure 7:**
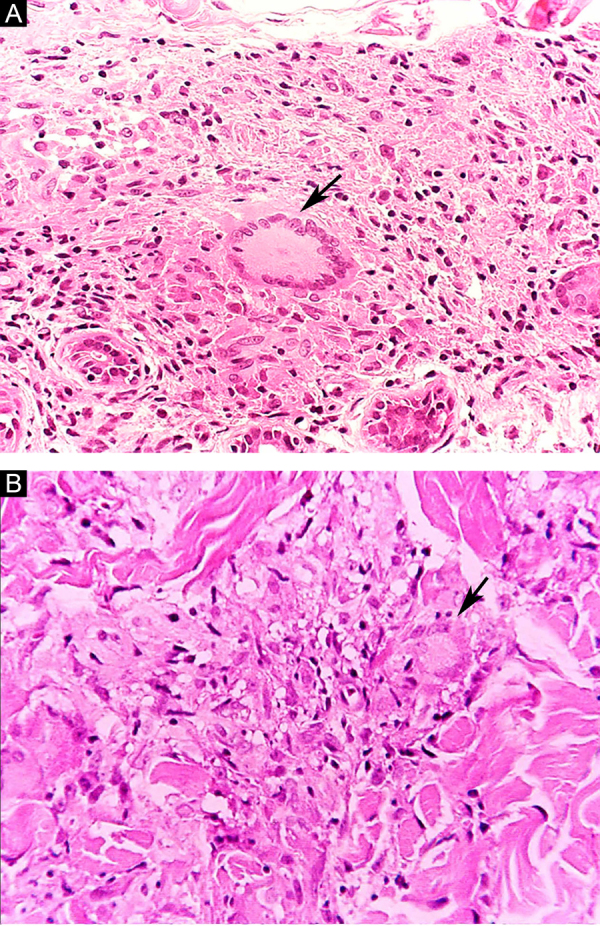
Tuberculoid leprosy. (A), Epithelioid granuloma with Langhans-type multinucleated giant cell (arrow). Borderline leprosy (B), Loose granulomatous arrangement with the presence of a multinucleated giant cell with fine cytoplasmic vacuolization (arrow). (Hematoxylin & eosin, ×400).

**Figure 8:**
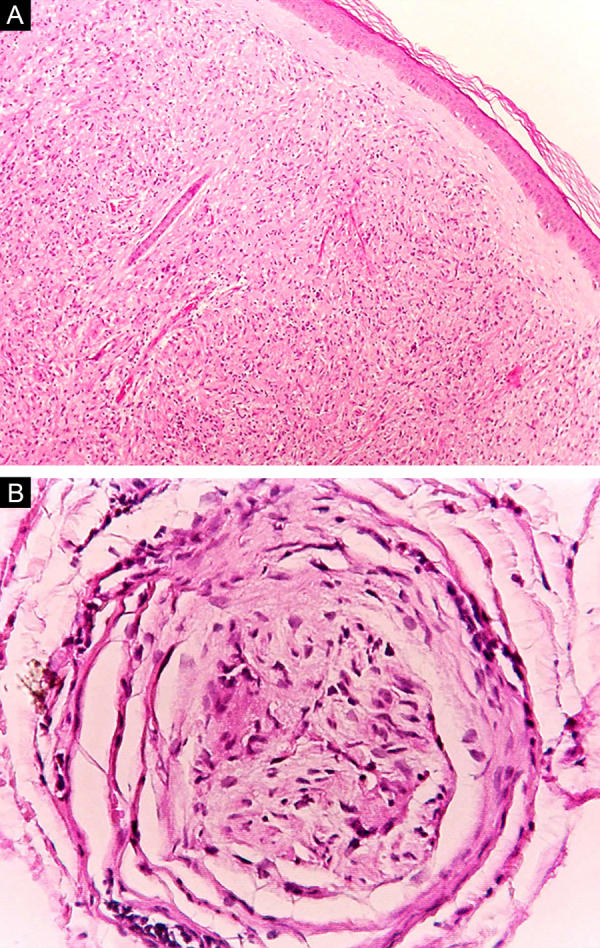
Lepromatous leprosy. (A), Rectification of the epidermis, which is separated from the dense dermal infiltrate by a collagen band (Hematoxylin & eosin, ×40). (B), Cross-section of a dermal nerve with perineural delamination (Hematoxylin & eosin, ×200).

Some key characteristics may be useful in the histopathological classification of leprosy. Epithelioid cells are present in the tissue response of TL, BT, and BB lesions. Langerhans cells are seen in TL and BT lesions (Fig. 7a). Vacuolated giant cells can be found in BB and BL lesions (Fig. 7b). Foamy macrophages are present in BL and LL lesions. The *grenz* zone (German term for "border") corresponds to a subepidermal area free of inflammatory infiltrate, which is observed in lesions of the LL, BL, and BB forms (Fig. 8a). On the other hand, lamination of the perineurium with the formation of an "onion bulb" is a typical finding of lesions of the LL and BL forms (Fig. 8b).^33^

**Figure 9:**
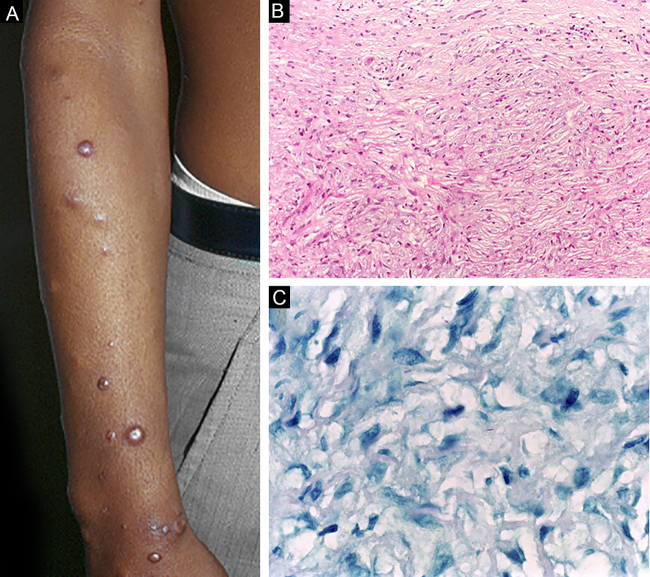
Histoid leprosy. (A), Well-delimited erythematous-ferruginous papules disseminated throughout the upper limb. (B), Proliferation of spindle-shaped macrophages with finely vacuolated cytoplasm (Hematoxylin-eosin, ×200). (C), Alcohol-acid resistant bacilli in the cytoplasm of spindle-shaped macrophages. (Faraco, ×1000).

Histoid leprosy, a variant of the lepromatous presentation, is a unique and uncommon entity with particular clinical and histopathological characteristics. The term histoid leprosy was originally coined by Wade in 1963 as a histopathological concept of leprosy rich in bacilli and consisting of spindle-shaped macrophages (Fig. 9b), together with the absence of globi formation. It is characterized by painless, firm, cutaneous, or subcutaneous papules and nodules covered by yellowish-brown skin, sometimes resembling dermatofibromas, LL lesions, or an ENL-like reaction (Fig. 9a). Histoid leprosy was classically associated with disease recurrence after monotherapy with dapsone in the presence of bacillus resistance to the drug; however, since the introduction of multidrug therapy, the majority of observed cases are "*de novo*" histoid leprosy.^34^

Leprosy in its initial presentation, called "indeterminate" leprosy, usually has a discrete clinical manifestation, represented by a small hypochromic macule with reduced sensitivity, and there may be hypohidrosis and/or thinning of hair at the lesion site. Histopathology shows only the presence of lymphohistiocytic infiltrate of perineural and periadnexal location, without the presence of bacilli. Indeterminate leprosy may progress to spontaneous cure or to any of the five classic presentations, depending on the immune response pattern developed by the host.^32^

Finally, the reactional episodes of leprosy correspond to acute immune exacerbations of the chronic infectious disease. The reversal reaction (RR) typically occurs in borderline patients, with the appearance of new lesions and worsening of pre-existing ones, with the development of erythema and edema over the lesions, which show hyperesthesia and become painful. The condition has a sudden onset, different from disease recurrence, which is slow and insidious. Neuritis is common, with loss of sensory or sensorimotor function and pain on palpation of the affected nerves, which are enlarged. Arthritis can occur, especially when there are skin lesions close to the joints.^37^ On histopathology, it is defined by the presence of two characteristics: granulomas with extra- and intracellular edema, dilated vascular channels, dermal collagen dissociation, evidence of intense late-type (IV) hypersensitivity response with acute damage to dermal nerves.^35^, ^36^

**Figure 10:**
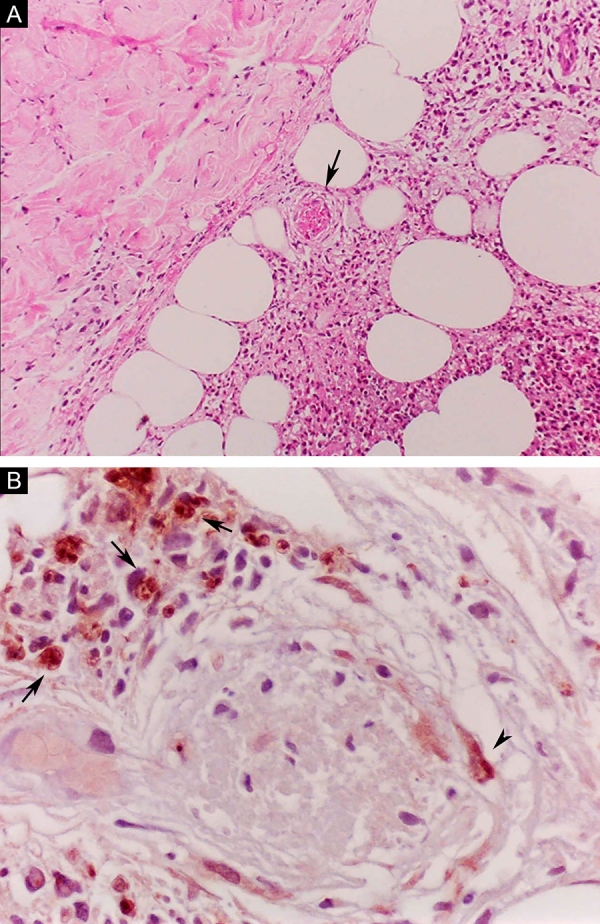
Erythema nodosum leprosum. (A), Involvement of hypodermal lobes with foci of neutrophilic exudation, macrophages, rupture of adipocytes, and blood vessel with thrombosis (arrow) (Hematoxylin & eosin, ×100). (B), Mycobacterial antigenic material in the cytoplasm of macrophages (arrows) and in endothelial cell (arrowhead) of a blood vessel with thrombosis (Immunohistochemical reaction with anti-BCG antibody, ×400).

Erythema nodosum leprosum (ENL), in turn, is diagnosed when a multibacillary patient (BB, BL, or LL) abruptly develops erythematous papulonodular lesions that are tender and tense to touch. In more severe cases, bullae formation and ulceration of the lesions may occur, characterizing necrotizing erythema nodosum. Unlike classic erythema nodosum, which is limited to the lower limbs, ENL tends to show a symmetrical distribution, affecting the face, trunk, and limbs, especially the extensor areas. More rarely, it may show a clinical picture that is identical to that of erythema multiforme, with erythematous plaques, purpuric lesions, and bullae. Neuritis, iritis, orchitis, dactylitis, adenopathy, edema, and fever may occur. Arthropathy is common, occurring in a variety of patterns, with the polyarticular pattern of small and large joints, similar to rheumatoid arthritis, being the most common.^37^ Significant visceral involvement may occur in the most severe cases, with an elevation of inflammatory tests (leukocytosis, ESR, CRP), liver dysfunction, anemia, and hematuria, and should be differentiated from the clinical picture of drug reactions and other diseases. On histopathology, it is characterized by a neutrophilic polymorphonuclear infiltrate foci, intermingled with a macrophage granulomatous response, edema, and often with evidence of vasculitis. The inflammatory process affects the dermis and hypodermis (Fig. 10).^36^

## Serological and molecular tests

Leprosy control strategies are still based on early clinical diagnosis and treatment only, aiming at blocking transmission and preventing, or at least minimizing, the sequelae. However, consistent with its characteristics of a neglected disease, it still does not benefit from sensitive and specific serological methods capable of assisting in the diagnosis and follow-up of patients. In this sense, some specific antibodies against *M. leprae* have been studied as potential markers, with special attention to phenolic glycolipid 1 (PGL-1)

Typically, multibacillary individuals produce large amounts of IgM anti-PGL-1 antibodies in an amount that is proportional to the bacterial load. In untreated patients, the mean sensitivity for multibacillary individuals is 78%, while paucibacillary patients have a sensitivity of only 23%. These figures allow the suggestion of positive serology usefulness for the diagnosis of suspected early multibacillary cases (especially primary neural ones, without skin lesions), as well as in recurrent reactional cases, in addition to the follow-up of multibacillary contacts.^38^, ^39^, ^40^ However, the serology is not commercially available, being limited to a few research centers, currently restricting its use in clinical practice.^37^, ^38^

The screening for IgM anti-PGL-1 antibody through a rapid test has been recommended in contacts of leprosy patients without characteristic clinical lesions, aiming at the early detection of the disease. A positive result, however, does not imply a diagnosis of leprosy since, in endemic areas, positive test results may reflect merely a subclinical infection.^40^, ^41^ Positive cases, in the absence of dermatological or neurological alterations, should be followed annually for the appearance of lesions. In the presence of suspected inconclusive alterations (dermatological or neurological) in contacts, the rapid test is recommended as an initial step, and positive cases should be further investigated through a bacilloscopic examination.^39^, ^40^

Finally, PCR is a molecular technique used to amplify the DNA of *M. leprae* with high accuracy, showing a sensitivity of 72% and a specificity of 87% for the multibacillary form. In paucibacillary patients, the sensitivity decreases to 45%, but specificity remains stable at 86%. The technique is particularly useful in clinical cases that are difficult to diagnose, with a negative bacilloscopy and inconclusive histopathology.^41^

## Conclusion

The clinical manifestations of leprosy are characterized by its bipolar spectral presentation, with a strong correlation between the most prominently activated arm of the immune system (Th1/Th2) and the clinical picture. The low genetic variability found in the *M. leprae* genome strongly suggests that the immune response depends mainly on the host and not on the pathogen variations. In fact, some genetic studies have already demonstrated a correlation between certain haplotypes and specific clinical manifestations, although the immunogenic determinants of the natural history of the disease are still far from being fully understood.

The basis of leprosy elimination and control has consisted mainly of improving access to health care, combined with a better capacity for screening and early diagnosis of cases. A Brazilian study indicated that patients start treatment, on average, 1.5 to 2 years after the onset of the first symptoms, with the delay being attributed, at least in part, to the limited capacity of health professionals to recognize the disease.^38^ In this sense, the wide dissemination of the current knowledge about the disease is an essential step to attain early diagnosis and treatment, control of reactions and sequelae, in addition to blocking the disease transmission – aspects that have been neglected in many parts of the world for a long time.

## Financial support

The authors are grateful for the financial support from *Fundo de Apoio à Dermatologia de São Paulo* – Sebastião Sampaio (FUNADERSP).

CNPq researcher grant: process n. 306371/2018-9 (research productivity grant to the author MNS).

## Authors' contributions

Luis Alberto Froes: Critical review of the literature; collection, analysis, and interpretation of data; drafting and editing of the manuscript.

Mirian Sotto: Approval of the final version of the manuscript; design and planning of the study; drafting and editing of the manuscript.

Maria Angela: Approval of the final version of the manuscript; design and planning of the study; drafting and editing of the manuscript.

## Conflicts of interest

None declared.
